# Frailty and Clinical Outcomes of Older Patients Admitted to an Emergency Department in Japan

**DOI:** 10.7759/cureus.74721

**Published:** 2024-11-29

**Authors:** Akifumi Maeda, Yousuke Tokoo, Yukari Konishi, Azusa Okura, Natsumi Imai, Yuko Tabuchi, Miyuki Sako, Katsuhiro Yorozu

**Affiliations:** 1 Nursing, Senri Kinran University, Osaka, JPN; 2 Nursing, Hokusetsu General Hospital, Osaka, JPN

**Keywords:** 75 years and older, emergency department, frail older adults, risk assessment tools, triage

## Abstract

Introduction

Medical advances and improved living standards have increased life expectancy, and the percentage of older adults is growing rapidly. The proportion of older adults visiting the emergency department (ED) is also increasing. Frailty is recognized as a significant risk factor for adverse outcomes. Thus, emergency nurses need to assess frailty in older patients presenting to the ED. This study aimed to investigate frailty and adverse outcomes among older adults who visited the ED.

Materials and methods

This was a prospective observational study. The study participants included patients aged 75 years and above who were either transported or self-presented to the ED of a secondary emergency medical institution at a community acute care general hospital. Data, including frailty, vital signs, triage levels, and other variables, were collected using the screener, an adverse outcome prediction tool. Mortality and survival groups of patients were compared.

Results

The mortality rate as assessed by the use of the adverse outcome prediction tool was significantly higher in the high-risk group than in the low-risk group (P = 0.018). Compared with outcomes in the survival group (n = 374, 95.4%), the 90-day mortality group (n = 18, 4.6%) showed significant differences in the scores, the need for assistance with housekeeping and bathing, and cognitive impairment. Regarding triage levels, no significant differences were observed in the screener-related items between the mortality and survival groups in the urgent category.

Conclusions

The results of this study showed no significant difference in 90-day mortality rates when comparing triage categories, suggesting the validity of assessing older adults with adverse outcome prediction tools. Therefore, beyond research facilities, during triage, the adverse outcome prediction tool can be used to assess the frailty of older adults, providing healthcare providers with the opportunity for early intervention in older adults who are frail.

## Introduction

Background

Medical advances and improved living standards have increased life expectancy, and the percentage of the aging population is rising rapidly. According to the United Nations, the number of older adults, especially those aged 75 and above, is expected to increase rapidly in the coming decades [1]. This demographic shift is expected to result in a significant increase in demand for older adults' healthcare services worldwide. The rate of emergency transport for older individuals, especially those above 75, is notably high in some countries due to the increased healthcare needs in this age group [2,3]. The problem is expected to become even more severe in the future.

With age, lean body mass decreases, and with the increase in sarcopenia, muscle strength and endurance decline, leading to physical, mental, and social vulnerabilities. Frailty is defined as a biological syndrome involving a state of decreased reserve and resilience to stressors as the result of cumulative decline in various physiological systems across the body, leading to increased frailty and adverse health outcomes [4]. Frailty, an age-associated predisposition to adverse healthcare outcomes [5], is increasingly prevalent. Frailty is also common among older emergency department (ED) visitors, with studies suggesting up to 60% are frail [6,7]. Identifying frailty at an early stage is important to triage people to appropriate care pathways [8]. Screening for frailty helps create awareness among healthcare professionals regarding the complexity of older adults, which leads to more holistic, person-centered care [9].

Frailty in older patients is a significant risk factor for adverse outcomes, such as mortality and long-term hospitalization [10]. However, because most frailty symptoms progress slowly, frailty can be easily overlooked in clinical practice or can simply be regarded as part of the aging process [11]. More than ever, healthcare providers are required to deepen their understanding of the characteristics of the aging population, such as frailty.

The frail older adults who are admitted to the ED have potential implications of longer waiting times to be seen by healthcare providers. These implications include an increased risk of intensive care unit (ICU) admissions due to delays in the administration of analgesics and antibiotics and an increased mortality rate [12]. In addition, frail older patients with dementia and comorbidities may develop dehydration and pain, which may lead to delirium while these patients are waiting to be seen [13]. Investigating the association between frailty and adverse outcomes, such as death, for older patients presenting to the ED is important due to the increasing number of older patients who will continue to visit the ED.

The study by Blomaard et al. [14] has shown that combining the assessment of urgency and frailty improves the predictability of early mortality in older patients. The healthcare providers must assess both acuity and frailty in older patients presenting to the ED to prevent adverse outcomes. The comprehensive geriatric assessment (CGA) has been referred to as the "gold standard" for frailty identification [15]. However, in EDs, due to system and patient pressures, time constraints, and reliance on specialist skills, performing CGAs routinely is not often feasible [16,17]. Therefore, it has been suggested that briefer validated scales be used to identify these high-risk patients who may still benefit from fewer CGAs [17]. A screening tool to be used among clinicians in the ED should be easily implementable, have the ability to score without relying on comprehensive patient documentation or equipment and be reproducible and sensitive to change over time.

Various instruments have been developed to assess frailty and predict adverse outcomes in older patients, including the Identification of Seniors at Risk Tool (ISAR) [18], Fried Frailty Scale [4], Stable, Unstable, Help to Walk, Bedbound (SUHB) Scales [19], and the Acutely Presenting Older Patient (APOP) screener [20]. In the ED, emergency nurses should use a simple screening tool to quickly assess older patients’ frailty [19]. Therefore, the APOP screener, which has high specificity and positive predictive value for mortality in older patients after admission to the ED and can be completed in about two minutes [21], was used in this study.

It is critical for emergency nurses to consider the decline in physical and cognitive functions and the physical, mental, and social vulnerabilities of older patients when assessing them in the ED. Appropriate assessment in the ED and early intervention after an ED visit may help to prevent adverse outcomes in this population.

Purpose

The purpose of this study was to investigate frailty and adverse outcomes among older adults who visited the ED in Japan.

## Materials and methods

Overview of the facility

The study facility was a secondary emergency medical institution, which is the community acute care hospital located in Osaka Prefecture, Japan. The ED provides medical and surgical care 24 hours a day and is staffed by three to four physicians, three to four nurses, and one to three paramedics, depending on the shift. In 2023, the ED accepted 3,519 emergency transport patients and 3,779 walk-in patients, a total of 7,298 patients. Of all patients, 3,445 (47.2%) were aged 75 and above. The facility utilizes the Japan Triage and Acuity Scale (JTAS), which is a validated, five-level triage system that is based on the CTAS and has been used nationwide [22].

Triage nurse

Triage nurses in the study facility must have at least three years of nursing experience and have completed in-hospital triage training. Of 31 ED nurses, 14 (45.2%) were qualified triage nurses.

APOP screener

The APOP screener is a screening instrument that assesses physical and cognitive functions in older patients aged 70 and older and predicts the risk of 90-day mortality and functional decline after an ED visit. The screener consists of the following seven items: (1) age, (2) sex, (3) use of emergency transport, (4) need for assistance in housekeeping or preparing meals, (5) need for assistance in bathing or showering, (6) hospitalization in the past six months, and (7) cognitive impairment. The screener calculates the rate (%) of 90-day mortality and functional decline risks. A risk percentage of 45% or more is classified as high risk, and less than 45% is classified as low risk [20].

Adverse outcomes

Frail older adults have an increased risk of long-term hospitalization, revisits to medical institutions, functional impairment, and death after visiting the ED. The post-visit mortality rate of older patients who visited the ED becomes stable only after 90 days of presentation [23]. In this study, we defined death within 90 days after an ED visit as an adverse outcome.

Study design

This was a prospective observational study.

Participants

The study participants included older adults aged 75 and above who were transported by ambulance or self-presented to the study facility's ED and were triaged using the JTAS triage tool in the urgent category (level 2), semi-urgent category (level 3), or less urgent category (level 4).

Exclusion

Patients triaged in the resuscitation category (level 1) were excluded from the study because life-saving interventions were prioritized over gathering information using the APOP screener. In addition, patients triaged in the non-urgent category (level 5) were excluded owing to the low need for emergency care. Patients with missing vital sign values or subjects with unknown outcomes were excluded.

Study period

The study data were collected for 16 months, from March 2023 to June 2024.

APOP form and implementation

Triage nurses used the APOP form, which contains seven items of the APOP screener, to collect data during triage. For item (1), age, we only collected data from patients aged 75 and above. For item (7), cognitive impairment, the triage nurses confirmed with family members or companions whether the patients had cognitive impairment rather than a disturbance of consciousness due to symptoms.

When patients could not appropriately respond to all or part of the screener questions because of their conditions, we asked their companions to provide the answers. After the patient’s assessment, the answers collected on the APOP form were entered into the APOP screener application [16] to obtain the 90-day mortality risk rate as a percentage and the risk level, high or low.

Data collection and analysis

The following data were collected: triage category assigned for the participant, the seven APOP screener items, outcome (discharge home, general admission, ICU admission, death within 90 days), and vital signs, including respiratory rate, peripheral arterial oxygen saturation (SpO2), blood pressure, pulse, and weight. Deaths within 90 days were verified in the patients’ electronic medical records or by contacting the patients or their families by phone. The data were saved in Excel (Microsoft Corporation, Redmond, WA, USA) with the password.

For nominal data, such as sex (female 0, male 1), emergency transport, assistance with housekeeping and preparing meals, assistance with bathing or showering, hospitalization in the past six months, cognitive impairment, death within 90 days after an ED visit, and admission, outcomes were converted to dummy variables (no, 0; yes, 1). For ratios, ordinal, and interval data, such as age, respiratory rate, SpO2, blood pressure, pulse, and body temperature, the Shapiro-Wilk test was used to confirm non-normality, and the data are presented as medians and interquartile ranges. SPSS Statistics version 26.0 (IBM Corp. Released 2019. IBM SPSS Statistics for Windows, Version 26.0. Armonk, NY: IBM Corp.) was used for analysis. The nominal data were categorized as yes or no and analyzed using chi-square tests, and the ratio, ordinal, and interval data were analyzed using the Mann-Whitney U test. The Kruskal-Wallis test was used to compare the three triage levels, and a significance level of 5% was used.

Ethical considerations

This study was conducted with the approval of the Ethics Committee of Hokusetsu General Hospital and with the permission of the hospital director (approval number: 2023-010). Participants were provided with an informed consent form at ED reception. After the research staff explained the study’s purpose and methods verbally and visually, participants gave their consent. The data sources, including emergency transport forms, medical questionnaires, triage forms, and electronic medical records, were reviewed in designated locations, and the data obtained were encrypted to ensure that individuals could not be identified. Additionally, the electronic data were password-protected and stored on a computer not connected to the Internet. Encrypted data were used in the publication to protect the participants' privacy. Data collected on paper forms will be stored securely in a locked cabinet for five years after the study completion and thereafter disposed of appropriately.

## Results

Data for analysis

Of 406 participants, data from 392 participants were analyzed, excluding 12 participants with missing vital sign data (eight with missing respiratory rates, two with missing SpO2, one with missing blood pressure, and two with missing body temperature) and one with an unknown outcome. Among them, 122 participants (31.1%) were triaged in the urgent category, 195 (49.7%) in the semi-urgent category, and 75 (19.1%) in the less urgent category. Death within 90 days after an ED visit was significantly higher in the APOP high mortality risk group (9.0%) than in the APOP low mortality risk group (2.8%) (P = 0.018; risk ratio (RR), 2.9) (Table 1).

**Table 1:** Comparison of participants and triage levels Note 1: The items marked with ^a^ represent nominal data, which lists the number of patients (percentages). Ratio, ordinal, and interval data are presented as medians (interquartile ranges). The percentage of ICU admissions was calculated based on the total number of patients admitted to the hospital after the ED visit. Note 2: The nominal data were analyzed using chi-square tests, and the ratio, ordinal, and interval data were analyzed using the Kruskal-Wallis test. Significant differences are indicated by P < 0.05*, P < 0.01**, and P < 0.001***. APOP: Acutely Presenting Older Patient, ICU: intensive care unit, ED: emergency department

Item	All participants (n = 392)	Urgent group (n = 122)	Semi-urgent group (n = 195)	Less urgent group (n = 75)	P-value
Sex (male) (%)^a^	180 (45.9)	59 (48.3)	89 (45.6)	32 (42.6)	0.734
Age (years)	84 (79-88)	84 (80-89)	84 (79-88)	84 (79-88)	0.639
Emergency transport (%)^a^	216 (55.1)	78 (63.9)	99 (50.7)	40 (53.3)	0.055
Assistance with housekeeping and meals (%)^a^	123 (31.3)	44 (36.0)	59 (30.2)	21 (28.0)	0.390
Assistance with bathing or showering (%)^a^	125 (31.8)	47 (38.5)	56 (28.7)	23 (30.6)	0.154
Hospitalization in the past 6 months (%)^a^	71 (18.1)	34 (27.8)	29 (14.8)	8 (10.6)	0.002**
Cognitive impairment (%)^a^	108 (27.5)	43 (35.2)	49 (25.1)	17 (22.6)	0.069
Total APOP scores (points)	1 (1-3)	3 (2-4)	1 (0-2)	1 (1-2)	0.080
Risk rate (%)	28 (20-48)	31 (21-53)	27 (19-45)	25 (19-45)	0.048*
High risk level (%)^a^	111 (28.3)	44 (36.0)	49 (25.1)	18 (24.0)	0.072
Adverse outcomes					
General admission (%)^a^	222 (56.6)	90 (73.7)	110 (56.4)	22 (29.3)	＜0.001***
ICU admission (%)^a^	21 (9.4)	14 (15.5)	6 (5.4)	1 (4.5)	0.001**
Death within 90 days (%)^a^	18 (4.5)	10 (8.1)	5 (2.5)	3 (4.0)	0.064
Chief complaint					
Respiratory symptoms (%)^a^	54 (13.8)	23 (18.9)	24 (12.3)	7 (9.3)	-
Cardiovascular symptoms (%)^a^	38 (9.7)	24 (19.7)	10 (5.1)	4 (5.3)	-
Gastrointestinal symptoms (%)^a^	106 (27.0)	32 (26.2)	52 (26.7)	22 (29.3)	-
Neurological symptoms (%)^a^	32 (8.2)	10 (8.2)	14 (7.2)	8 (10.7)	-
Trauma (%)^a^	92 (23.5)	6 (4.9)	65 (33.3)	21 (28.0)	-
Other (%)^a^	70 (17.9)	27 (22.1)	30 (15.4)	13 (17.3)	-

Comparison of patients in the 90-day mortality group and survival group

Eighteen participants (4.6%) died within 90 days after an ED visit. The mortality group had higher total APOP scores (P = 0.002), higher rates of APOP mortality risk (P = 0.010), and a higher proportion of APOP high-risk levels (P = 0.006, RR 3.3) compared with the survival group. Significant differences in the APOP screener items were found for assistance with housekeeping (P = 0.001, RR 4.7), assistance with bathing or showering (P = 0.001, RR 4.6), cognitive impairment (P = 0.006, RR 3.5), and general admission (P = 0.005, RR 6.5). A significant difference in vital signs was found for systolic blood pressure (P = 0.031), with the mortality group having significantly lower values than the survival group (Tables 2-3).

**Table 2:** Comparison of APOP items between the mortality group and survival group Note 1: The items marked with ^a^ represent nominal data, which lists the number of patients (percentages). Ratio, ordinal, and interval data are presented as medians (interquartile ranges). Note 2: The nominal data were analyzed using chi-square tests. Items with significant differences are shown with risk ratios and 95% confidence intervals. The ratio, ordinal, and interval data were analyzed using the Mann-Whitney U test. Significant differences are indicated by P < 0.05* and P < 0.01*. APOP: Acutely Presenting Older Patient, ICU: intensive care unit

	Mortality group (n = 18)	Survival group (n = 374)	P-value	Risk ratio (95% CI)
Sex (male) (%)^a^	7 (38.8)	173 (46.2)	0.632	-
Age (years)	84 (80-91)	84 (79-88)	0.494	-
Emergency transport (%)^a^	9 (50.0)	207 (55.3)	0.809	-
Assistance with housekeeping and meals (%)^a^	12 (66.6)	111 (29.6)	0.001**	4.7 (1.7-12.9)
Assistance with bathing or showering (%)^a^	12 (66.6)	113 (30.2)	0.001**	4.6 (1.6-12.6)
Hospitalization in the past 6 months (%)^a^	5 (27.7)	66 (17.6)	0.342	-
Cognitive impairment (%)^a^	10 (55.5)	98 (26.2)	0.006**	3.5 (1.3-9.1)
Total APOP scores (points)	3 (1-4)	1 (1-3)	0.002**	-
Risk rate (%)	49 (25-60)	27 (19-46)	0.010*	-
High risk level (%)^a^	10 (55.5)	101 (27.0)	0.006**	3.3 (1.2-8.8)
General admission (%)^a^	16 (88.8)	206 (55.0)	0.005**	6.5 (1.4-28.8)
ICU admission (%)^a^	3 (18.7)	18 (8.7)	0.165	-

**Table 3:** Comparison of vital signs between the mortality group and survival group Note 1: The data are presented as medians (interquartile ranges). The data were analyzed using the Mann-Whitney U test, and significant differences are indicated by P < 0.05*. SpO_2_: oxygen saturation

Item	Mortality group (n = 18)	Survival group (n = 374)	P-value
Respiratory rate (times/minute)	20 (18-22)	18 (17-20)	0.097
SpO_2 _(％)	96 (94-98)	97 (95-98)	0.218
Systolic blood pressure (mmHg)	130 (106-149)	141 (122-162)	0.031*
Diastolic blood pressure (mmHg)	79 (63-88)	81 (68-93)	0.227
Pulse rate (times/minute)	85 (72-102)	83 (71-95)	0.567
Body temperature (℃)	37.1 (36.2-37.8)	36.7 (36.4-37.3)	0.217

No significant difference in mortality was found between the three triage levels (P = 0.064). However, 10 participants (8.2%) in the urgent category, five (2.6%) in the semi-urgent category, and three (4.0%) in the less urgent category died. Comparison of the mortality and survival groups at each triage level was performed only for the urgent and semi-urgent categories, as the number of deaths in the less urgent category was insufficient for analysis (n = 3). In the urgent category, the mortality group had lower systolic blood pressure (P = 0.012) than did the survival group, but there was no clinically significant difference (115 (81-141) mmHg vs. 147 (122-168) mmHg). In the semi-urgent category, significant differences were found in the following APOP screener items: assistance with housekeeping (P = 0.028, RR 10.0), assistance with bathing or showering (P = 0.023, RR 10.9), and the proportion of APOP mortality risk (P = 0.035) (Tables 4-5).

**Table 4:** Comparison between mortality group and survival group in the urgent triage category Note 1: The items marked with ^a^ represent nominal data, which lists the number of patients (percentages). Ratio, ordinal, and interval data are presented as medians (interquartile ranges). The percentage of ICU admissions was calculated based on the total number of patients admitted to the hospital after the ED visit. The nominal data were analyzed using chi-square tests, and the ratio, ordinal, and interval data were analyzed using the Mann-Whitney U test. Significant differences are indicated by P < 0.05*. APOP: Acutely Presenting Older Patient, SpO_2_: oxygen saturation, ICU: intensive care unit, ED: emergency department

Item	Mortality group (n = 10)	Survival group (n = 112)	P-value
Sex (male) (%)^a^	4 (40.0)	55 (49.1)	0.745
Age (years)	81 (78-91)	84 (80-91)	0.525
Emergency transport (%)^a^	5 (50.0)	73 (65.1)	0.493
Assistance with housekeeping and meals (%)^a^	6 (60.0)	38 (33.9)	0.166
Assistance with bathing or showering (%)^a^	6 (60.0)	41 (36.6)	0.181
Hospitalization in the past 6 months (%)^a^	4 (40.0)	30 (26.7)	0.463
Cognitive impairment (%)^a^	6 (60.0)	37 (33.0)	0.163
Total APOP scores (points)	3 (1-4)	1 (1-3)	0.111
Risk rate (%)	44 (21-52)	30 (21-52)	0.336
High risk level (%)^a^	5 (50.0)	39 (34.8)	0.336
General admission (%)^a^	9 (90.0)	81 (72.3)	0.452
ICU admission (%)^a^	3 (33.3)	14 (17.2)	0.089
Respiratory rate (times/minute)	20 (19-23)	20 (18-22)	0.359
SpO_2 _(％)	96 (93-98)	96 (95-98)	0.734
Systolic blood pressure (mmHg)	115 (81-141)	147 (122-168)	0.012*
Diastolic blood pressure (mmHg)	80 (63-88)	86 (72-96)	0.283
Pulse rate (times/minute)	99 (66-115)	86 (70-105)	0.544
Body temperature (℃)	37.1 (36.0-38.0)	36.7 (36.4-38.1)	0.926

**Table 5:** Comparison between mortality group and survival group in the semi-urgent triage category Note 1: The items marked with ^a^ represent nominal data, which lists the number of patients (percentages). Ratio, ordinal, and interval data are presented as medians (interquartile ranges). The percentage of ICU admissions was calculated based on the total number of patients admitted to the hospital after the ED visit. Note 2: The nominal data were analyzed using chi-square tests. Items with significant differences are shown with risk ratios and 95% confidence intervals. The ratio, ordinal, and interval data were analyzed using the Mann-Whitney U test. Significant differences are indicated by P < 0.05*. APOP: Acutely Presenting Older Patient, SpO_2_: oxygen saturation, ICU: intensive care unit, ED: emergency department

Item	Mortality group (n = 5)	Survival group (n = 190)	P-value	Risk ratio (95% CI)
Sex (male) (%)^a^	2 (40.0)	87 (45.7)	1.000	-
Age (years)	81 (79-90)	84 (80-89)	0.137	-
Emergency transport (%)^a^	2 (40.0)	96 (50.5)	0.683	-
Assistance with housekeeping and meals (%)^a^	4 (80.0)	54 (28.4)	0.028*	10.0 (1.1-92.1)
Assistance with bathing or showering (%)^a^	4 (80.0)	51 (26.8)	0.023*	10.9 (1.1-99.8)
Hospitalization in the past 6 months (%)^a^	1 (20.0)	28 (14.7)	0.557	-
Cognitive impairment (%)^a^	10 (55.5)	46 (24.2)	0.598	-
Total APOP scores (points)	3 (1-4)	1 (1-3)	0.099	-
Risk rate (%)	44 (22-58)	30 (21-52)	0.035*	-
High risk level (%)^a^	2 (40.0)	101 (53.1)	0.070	-
General admission (%)^a^	5 (100.0)	105 (55.2)	0.070	-
ICU admission (%)^a^	0 (0.0)	6 (5.7)	1.000	-
Respiratory rate (times/minute)	20 (20-22)	20 (18-22)	0.458	-
SpO_2 _(％)	96 (94-98)	97 (95-98)	0.349	-
Systolic blood pressure (mmHg)	115 (88-137)	147 (122-167)	0.997	-
Diastolic blood pressure (mmHg)	80 (70-88)	86 (72-98)	0.822	-
Pulse rate (times/minute)	99 (69-113)	86 (70-105)	0.751	-
Body temperature (℃)	37.1 (36.0-38.0)	36.7 (36.4-38.1)	0.739	-

## Discussion

Geriatric Emergency Department Guidelines [24] recommend that emergency nurses assess the risk of adverse outcomes in older patients presenting to the ED soon after their arrival using ISAR [18] or a similar screening tool, regardless of the patient’s chief complaint. In this study, we used the APOP screener, which generates the rate of 90-day mortality risk after an ED visit as a percentage and a mortality risk level as high or low. The following discussion focuses on two themes derived from the results of this study: adverse outcomes and implications of the adverse outcome prediction tool in the older adults ED population.

Adverse outcomes

The main finding of this study was that regardless of triage level, older patients with a high risk in the tool had a three times higher 90-day mortality rate compared to patients who were identified as low-risk. These results were similar to those found in the study by Blomaard et al. [14], which included participants triaged in the urgent to less urgent categories. However, longer wait times in the ED might have led to adverse outcomes for patients triaged in the semi-urgent category because prolonged waiting times can delay the administration of analgesics and antibiotics, leading to an increase in ICU admissions and a rise in mortality rates [12]. Furthermore, additional studies are needed in the future that include waiting times.

Implications of the adverse outcome prediction tool in older adults in the ED

When comparing triage categories, we observed no significant differences in the 90-day mortality rate, suggesting that conventional emergency triage systems alone may not adequately capture frailty-related adverse outcomes in older adults. Therefore, ED triage for older adults should not only assess urgency but also evaluate the risk of adverse outcomes due to frailty. The use of standardized tools like the APOP screener enables emergency nurses to conduct quick and consistent risk assessments of adverse outcomes.

The mortality group was more likely to be admitted than the survival group, highlighting the importance of sharing the results of adverse outcome prediction tools between emergency and ward nurses to initiate interventions to prevent adverse outcomes in older adults immediately upon admission. Approximately 50% of patients transported by ambulance are discharged home [25], and emergency nurses provide discharge support to prevent revisits to the ED or emergency admission [24]. Among patients receiving discharge support, 73% were older adults aged 75 and above, with a significant number having cognitive impairment or being unable to perform ADLs independently [26]. In this study, the mortality group also had significantly more individuals with cognitive impairment and a need for assistance compared with the survival group. This suggests that emergency nurses must collaborate with medical social workers to ensure appropriate support is provided to older adults returning home. Therefore, healthcare providers need to develop detailed support measures and follow-up systems for older adults at high risk for adverse outcomes. Such measures can prevent adverse outcomes in this vulnerable aging population.

The results of this study showed no significant difference in 90-day mortality rates when comparing triage categories, suggesting the validity of assessing older adults with adverse outcome prediction tools. Therefore, at facilities of similar capacity, during triage, the adverse outcome prediction tool can be used to assess the frailty of older adults, providing healthcare providers with the opportunity for early intervention in older adults who are frail. The tool may also be used to assess frailty in the younger population visiting the ED.

Study limitations

The study data were collected from a single facility, limiting the generalizability of the results. In addition, the analysis between each triage category was conducted with a small sample size, making it impossible to obtain statistically significant results. Therefore, in future studies, it is necessary to collect data from different regions and multiple facilities to improve the reliability and validity of the findings. Further research should also focus on assessing the effects of interventions on older adults at high risk for adverse outcomes.

## Conclusions

This study used an adverse outcome prediction tool to investigate adverse outcomes in older adults aged 75 years and above who presented to the ED at a secondary emergency medical institution. Our findings included the following: The results of this study showed no significant difference in 90-day mortality rates when comparing triage categories, suggesting the validity of assessing older adults with adverse outcome prediction tools.
